# Enhancement of a modified Mediterranean-style, low glycemic load diet with specific phytochemicals improves cardiometabolic risk factors in subjects with metabolic syndrome and hypercholesterolemia in a randomized trial

**DOI:** 10.1186/1743-7075-5-29

**Published:** 2008-11-04

**Authors:** Robert H Lerman, Deanna M Minich, Gary Darland, Joseph J Lamb, Barbara Schiltz, John G Babish, Jeffrey S Bland, Matthew L Tripp

**Affiliations:** 1 Functional Medicine Research Center, MetaProteomics, LLC, 9770 44th Ave. NW, Ste 100, Gig Harbor, WA 98332, USA

## Abstract

**Background:**

As the worldwide dietary pattern becomes more westernized, the metabolic syndrome is reaching epidemic proportions. Lifestyle modifications including diet and exercise are recommended as first-line intervention for treating metabolic syndrome. Previously, we reported that a modified Mediterranean-style, low glycemic load diet with soy protein and phytosterols had a more favorable impact than the American Heart Association Step 1 diet on cardiovascular disease (CVD) risk factors. Subsequently, we screened for phytochemicals with a history of safe use that were capable of increasing insulin sensitivity through modulation of protein kinases, and identified hops *rho *iso-alpha acid and acacia proanthocyanidins. The objective of this study was to investigate whether enhancement of a modified Mediterranean-style, low glycemic load diet (MED) with specific phytochemicals (soy protein, phytosterols, *rho *iso-alpha acids and proanthocyanidins; PED) could improve cardiometabolic risk factors in subjects with metabolic syndrome and hypercholesterolemia.

**Methods:**

Forty-nine subjects with metabolic syndrome and hypercholesterolemia, aged 25–80, entered a randomized, 2-arm, 12-week intervention trial; 23 randomized to the MED arm; 26 to the PED arm. Forty-four subjects completed at least 8 weeks [MED (*n *= 19); PED (*n *= 25)]. All subjects were instructed to follow the same aerobic exercise program. Three-day diet diaries and 7-day exercise diaries were assessed at each visit. Fasting blood samples were collected at baseline, 8 and 12 weeks for analysis.

**Results:**

Both arms experienced equal weight loss (MED: -5.7 kg; PED: -5.9 kg). However, at 12 weeks, the PED arm experienced greater reductions (*P *< 0.05) in cholesterol, non-HDL cholesterol, triglycerides (TG), cholesterol/HDL and TG/HDL compared with the MED arm. Only the PED arm experienced increased HDL (*P *< 0.05) and decreased TG/HDL (*P *< 0.01), and continued reduction in apo B/apo A-I from 8 to 12 weeks. Furthermore, 43% of PED subjects vs. only 22% of MED subjects had net resolution of metabolic syndrome. The Framingham 10-year CVD risk score decreased by 5.6% in the PED arm (*P *< 0.01) and 2.9% in the MED arm (*P *< 0.05).

**Conclusion:**

These results demonstrate that specific phytochemical supplementation increased the effectiveness of the modified Mediterranean-style low glycemic load dietary program on variables associated with metabolic syndrome and CVD.

## Background

Overconsumption of a maladaptive, westernized diet consisting of foods that are calorie-dense, nutritionally-poor, phytochemical-depleted, highly processed and rapidly absorbable has been shown to increase systemic inflammation and reduce insulin sensitivity [1-3]. With chronic ingestion, this dietary pattern often results in metabolic syndrome (MetS), a physiologic state encompassing a cluster of metabolic abnormalities, including dyslipidemia, central obesity, hypertension, and glucose intolerance. These are all independent risk factors for the development of type 2 diabetes and/or cardiovascular disease (CVD) [4,5]. The NHANES 1999–2000 estimated the prevalence of MetS in the US at an unadjusted 34.5% or approximately 69 million persons [6]. As worldwide food consumption patterns shift to the aforementioned dietary pattern, MetS is becoming a significant burden in developing nations and global prevalence is growing [7,8].

It is widely viewed that MetS results from an increasing, perpetual state of whole body insulin resistance, which is strongly associated with dietary carbohydrate [9-11] and saturated fat [12], leading to high serum triglycerides (TG) and visceral adiposity [13-15]. Acute infusion of free fatty acids leads to the accumulation of TG in skeletal muscle and evokes whole body insulin resistance with the same temporal pattern [16-19]. Metabolites of lipid metabolism such as diacylglycerol have been shown to directly induce insulin resistance by chronically activating protein kinase C (PKC). PKC activation terminates insulin signaling, preventing crucial tyrosine phosphorylation by the insulin receptor, leading to impaired insulin signaling [14]. MetS is also associated with a state of chronic inflammation. Adipocyte leakage has recently been shown to result in the recruitment of macrophages, which envelope excess lipids, form foam cells, and release inflammatory cytokines, setting up a state of systemic, chronic inflammation [20,21]. These adipokines lead to the systemic activation of several protein kinases involved in inflammatory signal transduction, including phosphoinositide-3 kinase (PI3K), glycogen synthase kinase (GSK-3) and PKC that singly or in concert cause insulin resistance in skeletal muscle and adipose tissue [22-24]. Hence, therapies which reduce circulating lipids and reduce systemic inflammation have shown promise in the treatment of insulin resistance and MetS.

Lifestyle modifications including diet and exercise are recommended as first-line intervention for treating insulin resistance and MetS by the National Cholesterol Education Program (NCEP) and American Heart Association (AHA). The Mediterranean-style diet, high in plant foods and monounsaturated fatty acids and low in processed foods and refined carbohydrates, has been shown to reduce CVD risk factors [25-28] and inflammatory burden associated with MetS etiology [29]. Pharmacologic treatment is considered appropriate if the individual is refractory to a lifestyle approach [30,31]. A recent review of the literature reveals that both intensive lifestyle modifications and drugs such as rimonabant or rosiglitazone may reduce the prevalence of MetS in 25 – 33% of patients [32]. While new pharmacologic approaches to MetS are under development [7], relying on drug therapy for an epidemic caused by a maladaptive diet is not as rational as realigning dietary habits [33]. These findings suggest that new lifestyle modification approaches in the treatment of MetS and its complications should be important public health priorities.

Advancing knowledge in inflammation and insulin signaling suggest that reversing the chronic imbalances of these downstream kinases provides a promising and logical strategy for reducing insulin resistance and the metabolic abnormalities of MetS. Inhibition of downstream kinases could be accomplished by using pharmaceuticals such as sunitinib and imatinib (multi-target protein kinases drugs), approved to treat cancers but which may also cause remission of diabetes [34]. However, several dietary phytochemicals, such as genistein and curcumin, have been shown to be protein kinase inhibitors [35,36]. A recent study showed that combining additional dietary polyphenols with a Mediterranean diet could provide synergistic effects and positively impact postprandial dysmetabolism [33]. As such, an important aspect to consider in dietary recommendations for MetS is the incorporation of diverse, targeted biologically-active phytochemicals to address the multiple underlying mechanisms of MetS. In a previous study [37], we reported that the addition of soy protein and phytosterols to a Mediterranean-style, low glycemic load diet had a more favorable impact than the AHA Step 1 diet on cholesterol/HDL and TG/HDL, blood pressure (BP), and Framingham 10-year CVD risk score for coronary heart disease in overweight, postmenopausal, hypercholesterolemic women.

With the finding that soy protein and phytosterols plus a Mediterranean-style diet could favorably affect the TG/HDL, an indicator of MetS, we initiated a screening program to identify additional structurally diverse phytochemicals capable of increasing insulin sensitivity through the modulation of downstream kinases. We screened 203 botanical products in 3T3-L1 adipocytes and identified novel substituted 1, 3-cyclopentadiones as well as a number of proanthocyanidin extracts with adipogenic and anti-inflammatory activity. One of the substituted 1, 3-cyclopentadiones was *rho *iso-alpha acids (RIAA) derived from hops (*Humulus lupulus*). RIAA have been used as bitter flavoring agents in beer for decades. We found RIAA dose dependently inhibited GSK-3, PI3K, and PKCβ in cell free kinase assays (manuscript in preparation). Another bioactive material identified by our screen was the proanthocyanidin-rich extract (PAC) of *Acacia nilotica*. In addition to inhibiting GSK-3, PI3K, and PKC, it also inhibited IKKβ in a dose-dependent manner (manuscript in preparation). At 5:1 (RIAA:PAC), the greatest efficacy in reducing glucose and insulin in the *db/db *mouse model was observed (manuscript in preparation). Furthermore, in an unpublished human pilot study, MetS subjects who consumed a combination of RIAA and PAC showed greater lowering of fasting LDL, TG, and TG/HDL than the placebo group.

The objective of this study was to investigate whether by enhancing a modified Mediterranean-style, low glycemic load diet with specific phytochemical supplementation we could improve cardiometabolic outcomes in subjects with MetS. Targeted supplementation included soy protein, phytosterols, hops RIAA and acacia PAC. The same diet was used in both arms and incorporated a spectrum of unprocessed, modified Mediterranean-style foods with an overall glycemic load less than 65.

## Methods

### Subjects

Men and women between the ages of 25 to 80 years with MetS and hypercholesterolemia were recruited into this study. Inclusion criteria included body mass index (BMI) ≥ 27 kg/m^2^, TG ≥ 1.70 mmol/L and < 4.52 mmol/L, LDL ≥ 3.37 mmol/L, and at least 2 of the following 4 criteria: (i) waist circumference > 88 cm (women) and > 102 cm (men); (ii) HDL < 1.30 mmol/L (women), and < 1.04 mmol/L (men); (iii) BP ≥ 130/85 mm Hg and < 155/95 mm Hg or diagnosed hypertension on medication; and (iv) fasting glucose ≥ 5.55 mmol/L and ≤ 7.00 mmol/L. Initial screening of subjects' serum lipids and glucose was performed using an in-office device (Cholestech LDX^® ^System). Eligible subjects were further screened with complete metabolic profile and complete blood count. Some key exclusion criteria included involvement in a weight loss program leading to 10% or greater body weight loss over the preceding 6 weeks; use of blood glucose or cholesterol lowering medications or supplements, corticosteroid use in the preceding 12 weeks; NSAID use ≥ 3 days/week in the preceding 4 weeks; or a history of chronic illness. This study was approved by the Copernicus Group Independent Review Board and was conducted based on good clinical practice guidelines. Informed written consent was obtained from each participant before enrollment in the study.

### Study design

**Figure 1:**
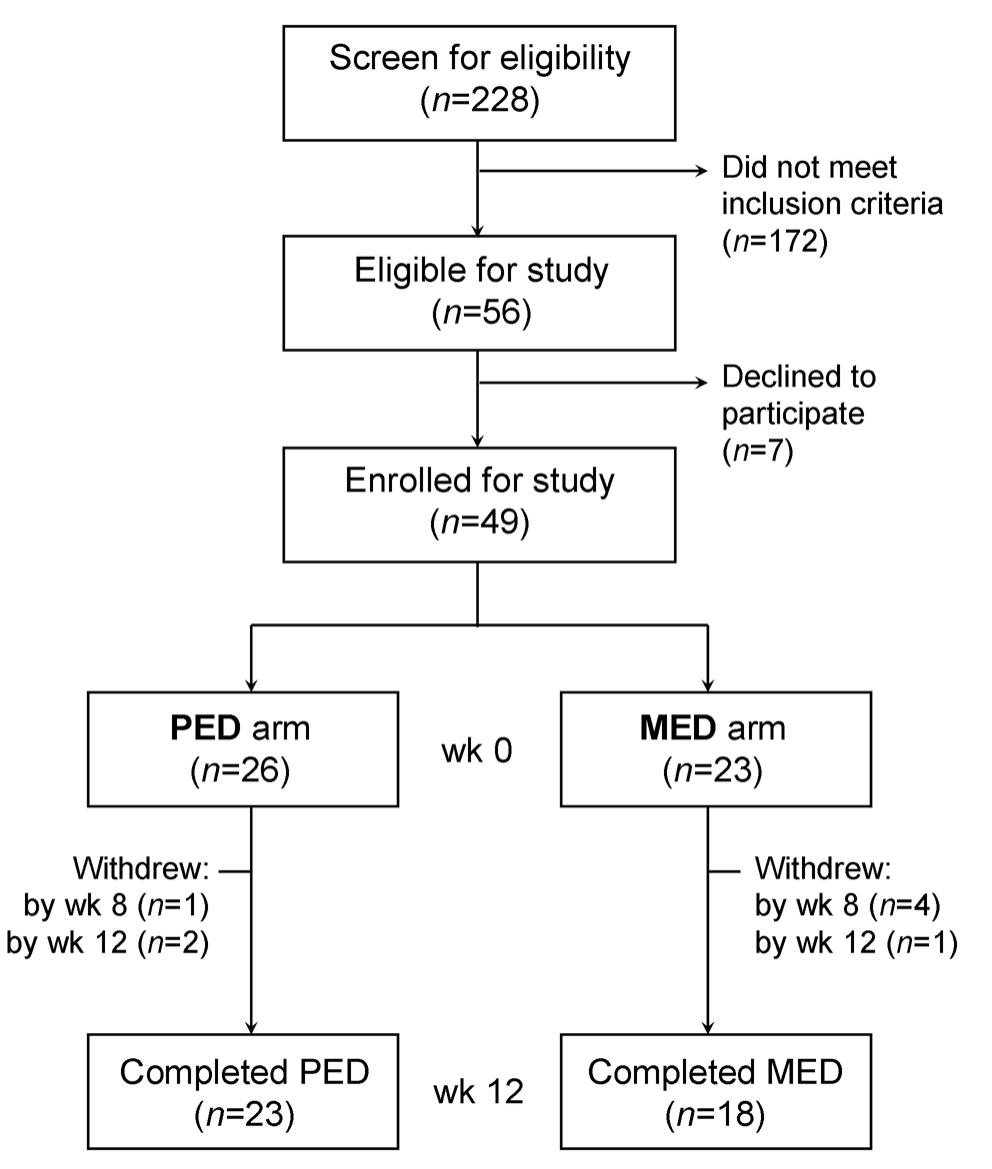
Flow chart of participant enrollment and study design.

This study was a randomized, 12-week, open-label, 2-arm trial conducted at the Functional Medicine Research Center in Gig Harbor, WA from June 15, 2006 through November 20, 2006 (Fig. 1). Subjects who satisfied the inclusion criteria were randomized to 1 of 2 arms using a commercial software program (Microsoft Excel^® ^2003; Microsoft); subjects were stratified by sex. Participants from both arms were instructed to follow a modified Mediterranean-style, low glycemic load diet (MED) and were provided with dietary guidelines, including a list of allowable foods, suggested serving sizes and recipes (see additional file 1: List of Permitted Foods and Beverages, Serving Sizes and Recipe). Subjects were asked to consume the diet until satisfied.

#### Modified Mediterranean-style low glycemic load diet (MED)

The rationale for defining this dietary program as "modified Mediterranean-style, low glycemic load" is that it includes a variety of low glycemic phytochemically-rich foods. Not all Mediterranean-style diets are low in glycemic load [38], and not all low glycemic load diets are phytochemically-diverse; thus, we chose to leverage the benefits of both by combining them together.

Specifically, the diet used in this study is distinguishable from the classic Mediterranean diet in that it is limited in the number of servings of alcohol and, in particular, whole grain. Alcohol intake was kept to a minimum: an optional 1 glass of red wine (4 oz) daily for all subjects. In the Mediterranean diet, several (typically 6 or more) servings of grain are often advocated; however, based on the available literature and our experience with this dietary program in the past decade, we decided to limit whole grains to 1 serving daily. Riccardi et al. [38] suggested that the standard Mediterranean diet may not be beneficial for individuals with insulin resistance due to the high carbohydrate content. Additionally, in our own use of this program, we found that reducing grain intake lowers cravings in many subjects. Moreover, Mediterranean-like food items such as pizza and hard toasted bread have been shown to have glycemic responses similar to white bread. Thus, one of the primary stipulations for foods in this dietary program was to ensure that all items included were low in glycemic load. The glycemic index of foods most commonly eaten was ≤ 55, with occasional selection (no more than 1 serving daily) from a small category of phytochemical-rich vegetables with a moderate glycemic index (55–70). This diet is also notable in that it omits all forms of sweeteners (natural and synthetic) except low-glycemic agave nectar syrup and stevia.

#### Phytochemical enriched diet (PED)

Subjects in the Phytochemical Enriched Diet (PED) arm additionally received (i) a combination of soy protein and plant sterols in a powdered beverage (2 servings/day, see additional file 2: Macronutrient profile of the soy and phytosterol-based powdered beverage) and (ii) a tablet containing RIAA from *Humulus lupulus L*. (RIAA magnesium salt) and PAC from *Acacia nilotica *bark (1 tablet, 2 times daily). Two daily servings of the powdered beverage (UltraMeal Plus^®^, Metagenics Inc.) provided 30 g of non-GMO soy protein (34 mg soy isoflavones) and 4 g of phytosterols (at least 40% β-sitosterol). The tablet contained 150 mg RIAA and 30 mg PAC. At 2 daily, subjects ingested a total of 300 mg RIAA and 60 mg PAC. Subjects were instructed to return all unused beverage powder and tablets, and the percentage of the amount consumed was calculated to indicate compliance. Subjects in the MED arm received neither the powdered beverage nor tablets. All participants were counseled to eat 3 meals/day plus snacks and to eat until hunger was satisfied. With or without the powdered beverage, the diet was designed to provide a total glycemic load of not more than 65. In addition to the diet, subjects in both arms were told to exercise aerobically, for a goal of 150 minutes/week, at 50–75% of maximum heart rate. Individual and target heart rates were calculated for each subject, and instruction on monitoring heart rates was provided.

#### Phytochemical tablet description

##### RIAA from *Humulus lupulus L *(150 mg/tablet)

**Figure 2:**
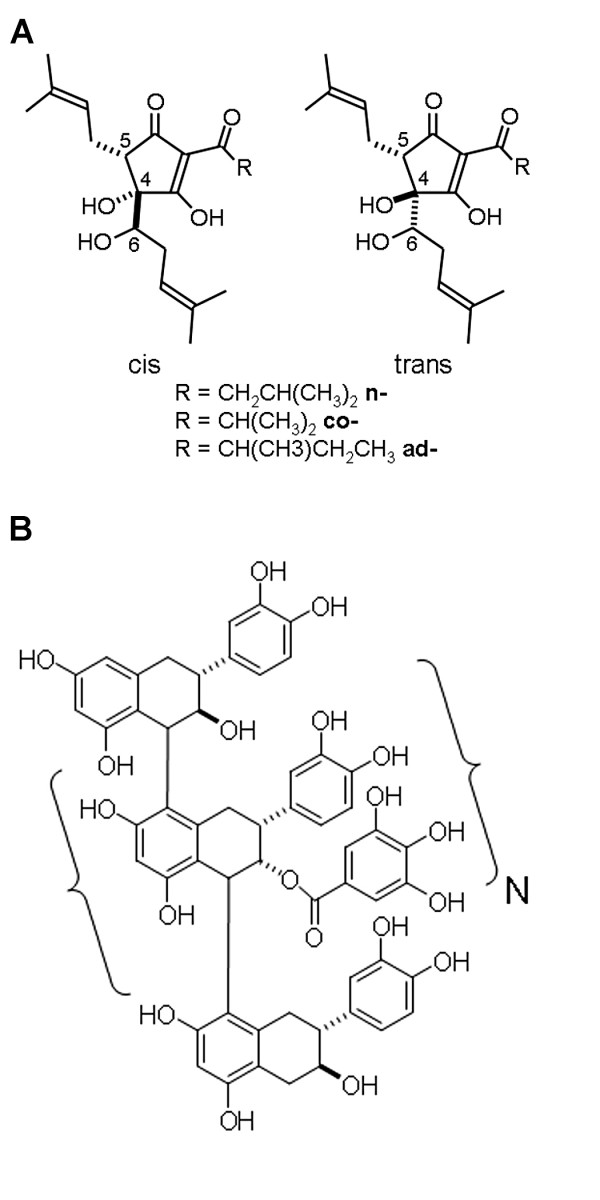
**Structural formula of (A) *rho *iso-alpha acids from *Humulus lupulus L*. and (B) proanthocyanidins from *Acacia nilotica *bark extract (N = 4–10 for oligomeric, and > 10 for polymeric fractions).*** rho *iso-alpha acids formulation contains approximately 25% inorganic salts, eg., Mg^+2^, K^+^, and SO_4_^-2^, 5% low-molecular resin and 68% total *rho *iso-alpha acids, as a mixture of related analogs and diastereomers with a ratio of *cis *to *trans *of 3:1 and a ratio of co- to n- of 1:2.2. The aqueous/methanol acacia extract consists of approximately 16% small molecule catechins and gallates, 28% oligomeric proanthocyanidins with a degree of polymerization of 4–8 and 56% polymeric proanthocyanidins with a degree of polymerization of 58.

For this study, a commercial preparation of the dried RIAA magnesium salt (Mg-RIAA) provided by John I. Haas (Yakima, WA) was used. As supplied, this material contained approximately 25% inorganic salts, eg., Mg^+2^, K^+^, and SO_4_^-2^, 5% low-molecular resin and 68% total RIAA. This RIAA fraction is a well-characterized mixture of related analogs and diastereomers (Fig. 2) with a ratio of *cis *to *trans *of 3:1 and the ratio of co- to n- of 1:2.2. The rationale for the magnesium salt form of the extract is that it provides a free flowing powder allowing for the blending and tablet manufacture of the finished product.

##### PAC from *Acacia nilotica *bark (30 mg/tablet)

Acacia PAC extract was provided by KDN-Vita International/Indfrag Ltd. Due to the manufacturing process, the extract has a great deal of chemical homogeneity. The chemical variation relates more to the level of polymerization (Fig. 2) than differences in the monomers, which are mostly catechins and gallates. Generally, an aqueous/methanol acacia extract consists of approximately 16% small molecule catechins and gallates, 28% oligomeric PAC and 56% polymeric PAC.

### Measurements

After the baseline visit, subjects returned at 2, 4, 8, and 12 weeks for follow-up visits. At each visit, 3-day diet diaries (including food choice, amount, time eaten) and 7-day exercise diaries (including form of exercise, intensity, duration) were evaluated and subjects were counseled on compliance to diet and exercise goals. Dietetic food models (Nasco) were used for accurate estimation of food intake. Data from 3-day diet diaries were analyzed using Genesis R&D 6.30 (ESHA Research). Glycemic load was calculated as described previously [39]. Body weight and BP were measured at each visit. BP was measured with an automatic BP monitor (Model HEM-711, Omron Healthcare, Inc.). Waist circumference was measured at the narrowest point between the iliac crest and the lowest rib at baseline, at 8 weeks, and 12 weeks.

Subjects completed a Food Craving Inventory (Pennington Biomedical Research Center), a Medical Outcome Study Short Form 36 (a questionnaire to assess the quality of life) [40], and a satiety questionnaire at each visit. The Food Craving Inventory listed 28 different foods and instructed the subject to rate cravings to consume each particular food over the preceding month [41]. The questionnaire was scored according to groupings of foods in 4 subscales: high fats, sweets, carbohydrates/starches, and fast foods. A higher score indicated increased levels of craving. Satiety was assessed using a 10 cm visual analog scale in which subjects were asked to assess feelings of hunger since their last visit at three different times during the day; a higher score indicated more feelings of hunger. Individual scores were averaged for overall satiety per subject. Diet and exercise compliance were assessed at each visit by one of the investigators. The number of minutes of aerobic exercise was obtained from each subject's 7-day exercise diary. The Framingham 10-year CVD risk score was calculated as described previously [42] by using age and relevant laboratory and questionnaire data for every individual.

### Laboratory analyses

Following an overnight fast, blood samples were collected from subjects at baseline, 8 weeks, and 12 weeks and stored at -80°C. Analysis of serum samples was conducted in batches and, except for lipoprotein subclass analysis, was performed by Laboratories Northwest. Glucose, lipids, and complete metabolic profiles of serum samples were assayed using a Vitros 950IRC analyzer (Ortho-Clinical Diagnostics). LDL was determined indirectly using the Friedewald formula: LDL = total cholesterol – HDL – TG/5 [43]. Non-HDL was determined by subtracting HDL from total cholesterol [44]. Apolipoproteins (apo) A-I and B were analyzed by turbidimetry using an Advia 1650^® ^(Bayer Diagnostics). Lipoprotein subclass particle analysis was done with an automated NMR spectroscopic assay by LipoSciences, Inc. Insulin was determined by a chemiluminescent, immunometric assay using the DPC Immulite 2000 (Diagnostics Products Corporation). HbA1c was quantified on fresh blood samples by ion exchange HPLC (Bio-Rad Variant II). Complete blood count was done on fresh blood by standard laboratory methods.

### Statistical analysis

Sample size was determined based on the results of an earlier study [37] in which a mean decrease of 0.62 mmol/L in LDL with the SD of 0.85 mmol/L was reported. Assuming a significance level of 0.05 with the power of 80%, a sample size of 17 subjects per treatment group was needed. We recruited more than 34 subjects to account for possible attrition. The data were analyzed as follows: for each variable, changes from baseline to 8 weeks and 12 weeks were calculated for each treatment group. Baseline determinations were analyzed using 2-sided t-tests. Changes from baseline to 8 weeks and 12 weeks were analyzed separately for each arm using *a priori *one-sided paired t-test. To detect any treatment differences between the arms, one sided unpaired t-tests were used. Additional analyses adjusting for calorie intake change, carbohydrate intake change and body weight loss were performed by using General Linear Model. Two-sided Wilcoxon signed-rank analysis was used to determine the significance of change in MetS score within arms. Missing values were not imputed for these analyses. Data were reported as means ± SE and analyzed using SAS (software version 8.1, SAS Institute). The probability of a type I error was set at the nominal 5 percent level.

## Results

### Subjects

The characteristics of subjects at baseline are presented in Table 1. None of the subjects had been in a weight loss program over the preceding 6 weeks. Of the 26 subjects randomized to the PED arm, 25 completed 8 weeks and 23 completed the trial; of the 23 subjects assigned to the MED arm, 19 completed 8 weeks and 18 completed the trial. Among those who did not complete the trial, all but 1 withdrew due to personal issues (e.g. traveling distance); one subject reported intolerance to the powdered beverage. Approximately two-thirds of the subjects (66%) were women. The average age of the participants was 53 years, and 82% of the subjects were obese (BMI ≥ 30). Overall, both arms were well matched with respect to the initial variables. However, the mean baseline HbA1c was higher in MED than PED subjects (6.04% vs. 5.70%, *P *= 0.02). The complete blood count and complete metabolic profile variables for all subjects remained stable within the reference ranges (data not shown). One subject in each arm was accepted outside of study criteria meeting 2 rather than at least 3 components of MetS. Both were women; one in the MED arm with borderline waist circumference of 34 inches; the other in the PED arm with borderline TG 1.63 mmol/L. In addition, at baseline, two other subjects in the PED arm no longer met MetS criteria.

**Table 1:** Baseline characteristics of study participants^1^

	PED arm	MED arm	*P *value
*n*^2^	25	19	
Sex			
Men	9	6	
Women	16	13	
			
Age, *year*	53.3 ± 2.4	51.7 ± 2.1	0.65
Wt, *kg*	95.3 ± 3.8	99.6 ± 3.6	0.42
Waist, *cm*	106.9 ± 2.0	111.6 ± 3.0	0.18
BMI, *kg/m*^2^	33.2 ± 0.8	35.2 ± 0.9	0.09
BP, *mmHg*			
Systolic	129.7 ± 3.5	132.8 ± 3.6	0.54
Diastolic	87.0 ± 2.3	85.2 ± 2.0	0.57
Fasting glucose, *mmol/L*	5.49 ± 0.10	5.63 ± 0.11	0.40
Fasting insulin, *pmol/L*	82.65 ± 8.33	110.43 ± 13.13	0.07
HbA1c,*%*	5.7 ± 0.1	6.0 ± 0.1	0.02
Cholesterol, *mmol/L*	6.57 ± 0.15	6.67 ± 0.20	0.71
LDL, *mmol/L*	4.39 ± 0.17^3^	4.64 ± 0.20^4^	0.34
TG, *mmol/L*	2.78 ± 0.22	2.37 ± 0.23	0.21
HDL, *mmol/L*	0.98 ± 0.05	1.02 ± 0.05	0.65
TG/HDL	3.07 ± 1.7	2.50 ± 1.4	0.23

^1^Values for continuous variables are means ± SE

^2^Subjects completing at least 8 weeks of study

^3^Data from 23 subjects

^4^Data from 17 subjects

### Caloric and macronutrient intake, study compliance, and food craving

No differences between the groups were noted at baseline in dietary caloric or macronutrient intake (Table 2), and taking into account supplementation, the glycemic load did not differ between arms at 8 weeks and 12 weeks. The total caloric intake and daily fat consumption, especially the saturated fatty acid, declined in both arms but the difference between arms at 8 weeks and 12 weeks was not significant. Monounsaturated fatty acid intake decreased in both arms compared to their baseline, but the percent energy from monounsaturated fatty acids remained unchanged throughout the study (10.0% at baseline and 10.2% at 12 weeks for PED; 10.8% at baseline and 11.2% at 12 weeks for MED). The carbohydrate intake decreased in both arms over time. The intake of soluble fiber increased 5-fold in the PED arm at 8 weeks and 12 weeks but only 1.5-fold in the MED arm. Daily consumption of protein by PED subjects increased over time (~16% increase by 12 weeks) whereas it decreased in the MED subjects (~13% decrease by 12 weeks). Alcohol consumption declined in both arm but the difference between arms at 8 weeks and 12 weeks was not significant.

**Table 2:** Caloric and nutrient intake at baseline, 8 weeks and 12 weeks

Nutrient	Visit	PED arm^1^	MED arm^2^	*P *value (between arms)
		*mean ± SE*		
Total energy (*kJ/d*)	Baseline	9654 ± 571	11575 ± 1619	0.22
	8 weeks	6699 ± 370	6171 ± 479	0.38
	12 weeks	6442 ± 285	5835 ± 472	0.25
Carbohydrates (*g/d*)	Baseline	261.7 ± 19.0	337.9 ± 45.8	0.10
	8 weeks	188.9 ± 10.9	150.9 ± 13.8	**0.03**
	12 weeks	169.6 ± 9.3	151.4 ± 13.6	0.22
Insoluble fiber (*g/d*)	Baseline	6.0 ± 0.7	4.3 ± 0.6	0.09
	8 weeks	10.4 ± 1.3	8.1 ± 1.0	0.17
	12 weeks	9.4 ± 1.0	8.3 ± 1.4	0.51
Soluble fiber (*g/d*)	Baseline	2.3 ± 0.3	2.1 ± 0.3	0.58
	8 weeks	11.8 ± 0.5	3.1 ± 0.4	**< 0.01**
	12 weeks	11.5 ± 0.3	3.3 ± 0.5	**< 0.01**
Protein (*g/d*)	Baseline	100.2 ± 7.0	97.7 ± 13.0	0.86
	8 weeks	112.5 ± 6.1	90.7 ± 7.2	**0.03**
	12 weeks	116.1 ± 7.6	85.3 ± 7.9	**0.01**
Fat (*g/d*)	Baseline	96.2 ± 7.4	113.5 ± 17.1	0.32
	8 weeks	47.6 ± 3.7	59.5 ± 5.18	0.06
	12 weeks	46.8 ± 4.7	52.7 ± 5.19	0.40
Saturated fatty acid (*g/d*)	Baseline	30.5 ± 2.9	35.0 ± 4.5	0.39
	8 weeks	10.5 ± 0.9	14.0 ± 1.7	0.05
	12 weeks	10.1 ± 1.1	12.8 ± 1.3	0.11
Monounsaturated fatty acid (*g/d*)	Baseline	25.6 ± 3.1	33.1 ± 5.5	0.21
	8 weeks	14.8 ± 1.5	19.7 ± 1.7	**0.04**
	12 weeks	17.4 ± 2.2	17.4 ± 2.3	0.98
Cholesterol (*mmol/d*)	Baseline	9.48 ± 0.96	9.22 ± 1.10	0.86
	8 weeks	6.31 ± 0.69	10.01 ± 1.38	**0.01**
	12 weeks	6.79 ± 0.94	8.33 ± 1.03	0.34
Alcohol (*g/d*)	Baseline	4.9 ± 1.4	3.3 ± 2.3	0.56
	8 weeks	1.2 ± 0.8	3.1 ± 1.2	0.19
	12 weeks	1.6 ± 0.9	2.1 ± 1.3	0.75

^1^PED arm: Baseline & 8 weeks, *n *= 25; 12 weeks, *n *= 23. ^2^MED arm: Baseline & 8 weeks, *n *= 19; 12 weeks, *n *= 18.

With respect to overall exercise compliance, the two arms were well matched at baseline, and compliance did not change over the course of the trial according to evaluation of the 7-day exercise diaries. Dietary compliance, as evaluated by glycemic load, did not differ at either 8 weeks or 12 weeks between groups, and glycemic loads of both arms were < 65. Compliance for the supplementation in the PED arm was high, with 93% for the powdered beverage and 95% for the phytochemical tablet. Craving for fast foods was greater at baseline in the MED (*P *= 0.03) than the PED arm. Cravings decreased significantly from baseline for sweets, fast foods, fats, and carbohydrates by 2 weeks and remained significantly reduced in both arms throughout the 12 weeks of study. There were no statistical differences between the arms from 2 weeks through 12 weeks. Also, hunger/satiety did not differ at baseline between the arms. Hunger between the three major meals (breakfast, lunch, and dinner), and general hunger throughout the day decreased from baseline to 8 and 12 weeks in subjects on both arms. However, hunger between the end of the evening meal and retiring decreased significantly only in the PED arm at both 8 and 12 weeks (-1.88 ± 0.39, *P *< 0.01, and -1.63 ± 0.47, *P *< 0.05, respectively), but not in the MED arm (-0.49 ± 0.56 and -0.82 ± 0.57, respectively). No differences between the arms were noted in quality-of-life questionnaire form Medical Outcome Study Short Form 36 (data not shown).

### Weight loss, waist circumference, and BP

Although this was not designed to be a weight-loss study, subjects in both arms who completed the 12-week study lost nearly identical amounts of weight (PED arm, -5.9 ± 0.7 kg; MED arm, -5.7 ± 1.0 kg). Similarly, in both groups, waist circumference was reduced significantly (*P *< 0.01) at 8 and 12 weeks. With regard to BP, subjects in the PED arm experienced reductions in both systolic and diastolic BP at the end of the trial; from 129.7 ± 3.5 mm Hg at baseline to 123.4 ± 2.7 mm Hg at 12 weeks for systolic BP (*P *= 0.025); and from 87.0 ± 2.3 mm Hg at baseline to 82.0 ± 2.0 mm Hg at 12 weeks for diastolic BP (*P *= 0.005). Subjects in the MED arm, on the other hand, displayed a decline only in systolic BP (132.8 ± 3.6 mm Hg at baseline to 128.1 ± 1.8 mm Hg at 12 weeks, *P *= 0.03); their diastolic BP remained unchanged throughout the study (85.2 ± 2.0 mm Hg at baseline to 84.4 ± 1.6 mm Hg).

### Serum lipids and apolipoproteins

**Figure 3:**
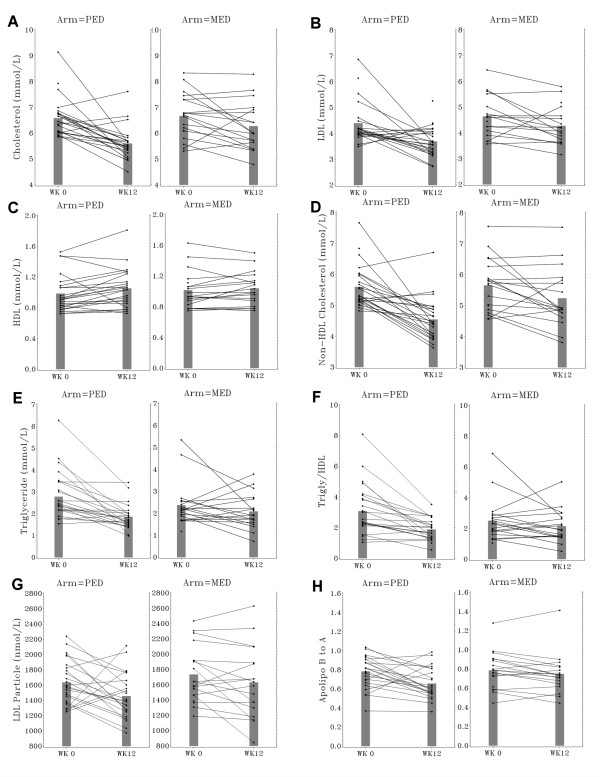
**Individual responses in serum lipid parameters (A, total cholesterol; B, LDL; C, HDL; D, non-HDL cholesterol; E, TG; F, TG/HDL; G, LDL particle number; H, apo B/apo A-I) in the intervention (PED) and control (MED) arms at baseline and 12 weeks.** Shaded bars indicate mean responses.

Effects of intervention diets on cardiometabolic risk variables are summarized in Table 3 and individual responses are illustrated in Fig. 3. Subjects in both arms experienced reductions in total cholesterol, LDL, non-HDL cholesterol, and cholesterol/HDL at 8 weeks and 12 weeks of the trial. The decrease in total cholesterol and non-HDL cholesterol from baseline to 12 weeks was greater in the PED than in the MED arm (*P *= 0.03 and 0.02, respectively): the average percent reduction in total cholesterol was 14.5% in the PED arm but only 6.3% in the MED arm and in non-HDL cholesterol was 18.2% in the PED arm but only 8.0% in the MED arm. The decrease in cholesterol/HDL was greater in the PED than in the MED arm (*P *= 0.01). With regard to serum TG and TG/HDL, a recognized marker of MetS, subjects in both arms experienced reductions at 8 weeks. However, only subjects in the PED arm continued to exhibit decreases in serum TG and TG/HDL at 12 weeks; the difference between the 2 arms was significant at 12 weeks (*P *= 0.03 and 0.02, respectively). There was an average 42.7% reduction in TG/HDL in the PED arm and only a 17.6% decrease in the MED arm. Serum HDL increased from baseline to 12 weeks only in subjects on the PED arm (*P *< 0.05), and remained unchanged in the MED arm. Compared to baseline, the total LDL particle number decreased in the PED arm at 8 and 12 weeks, and in the MED arm only at 12 weeks. VLDL particle number decreased at both 8 and 12 weeks only in the PED arm; large HDL particle number increased in both arms at 8 and 12 weeks (data not shown). With respect to lipoprotein variables, both PED and MED arms showed reduction of apo B concentration and apo B/apo A-I at 8 weeks compared to baseline; however, only the PED arm exhibited continued reduction in both variables from 8 weeks to 12 weeks. Additional adjustment for calorie and carbohydrate intake, and body weight change for these variables did not change the findings (adjusted P values in Table 3).

**Table 3:** Effect of intervention diets on cardiovascular risk variables at baseline, 8 weeks and 12 weeks of intervention

Variable	Visit	PED arm		MED arm		P value^2 ^(Difference between arm)	Adjusted P value^3^
					
		Value (*mean ± SE*)	Mean % change from baseline	Value (*mean ± SE*)	Mean % change from baseline		
Cholesterol (*mmol/L*)	Baseline	6.57 ± 0.15		6.67 ± 0.20			
	8 weeks	5.67 ± 0.22**	-13.8%	6.09 ± 0.25**	-8.7%	0.13	**0.04**
	12 weeks	5.59 ± 0.14**	-14.5%	6.27 ± 0.22*	-6.3%	**0.03**	**0.04**
TG (*mmol/L*)	Baseline	2.78 ± 0.22		2.37 ± 0.23			
	8 weeks	2.02 ± 0.25**	-27.2%	1.84 ± 0.17**	-22.3%	0.27	0.05
	12 weeks	1.86 ± 0.12**	-35.2%	2.09 ± 0.19	-14.3%	**0.03**	**0.01**
HDL (*mmol/L*)	Baseline	0.98 ± 0.05		1.02 ± 0.05			
	8 weeks	0.99 ± 0.05	0.7%	1.00 ± 0.04	-1.6%	0.34	0.45
	12 weeks	1.05 ± 0.05*	7.0%	1.04 ± 0.05	2.7%	0.18	0.21
LDL (*mmol/L*)	Baseline	4.39 ± 0.17		4.64 ± 0.20			
	8 weeks	3.78 ± 0.19**	-16.8%	4.24 ± 0.20**	-9.8%	0.11	0.19
	12 weeks	3.69 ± 0.12**	-17.3%	4.28 ± 0.17*	-8.4%	0.11	0.18
Non-HDL cholesterol (*mmol/L*)	Baseline	5.59 ± 0.14		5.65 ± 0.19			
	8 weeks	4.67 ± 0.20**	-16.4%	5.09 ± 0.25**	10.0%	0.19	**0.03**
	12 weeks	4.54 ± 0.14**	-18.2%	5.23 ± 0.22*	-8.0%	**0.02**	**0.03**
LDL particle number (*nmol/L*)	Baseline	1634 ± 57		1733 ± 84			
	8 weeks	1453 ± 63*	-11.9%	1618 ± 108	-6.6%	0.24	0.19
	12 weeks	1455 ± 63*	-11.9%	1629 ± 109*	-7.0%	0.26	0.31
Chol/HDL	Baseline	6.9 ± 0.3		6.8 ± 0.3			
	8 weeks	5.9 ± 0.3**	-14.3%	6.2 ± 0.3**	-9.0%	0.13	0.09
	12 weeks	5.6 ± 0.3**	-19.5%	6.3 ± 0.4**	-8.9%	**0.01**	**0.02**
TG/HDL	Baseline	3.1 ± 1.7		2.5 ± 1.4			
	8 weeks	2.1 ± 1.3**	-30.6%	1.9 ± 0.9**	-23.2%	0.20	0.07
	12 weeks	1.9 ± 0.7**	-42.7%	2.1 ± 1.1	-17.6%	**0.02**	**0.01**
apo A-I (*g/L*)	Baseline	1.94 ± 0.06		1.96 ± 0.07			
	8 weeks	1.89 ± 0.05	-2.5%	1.88 ± 0.06	-3.9%	0.32	0.30
	12 weeks	1.90 ± 0.06	-2.1%	1.87 ± 0.06	-4.3%	0.24	0.16
apo B (*g/L*)	Baseline	1.48 ± 0.05		1.50 ± 0.06			
	8 weeks	1.30 ± 0.05**	-12.1%	1.36 ± 0.06**	-9.9%	0.32	0.21
	12 weeks	1.21 ± 0.04**	-17.5%	1.36 ± 0.06**	-9.9%	0.09	0.14
apoB/apoA-I	Baseline	0.78 ± 0.03		0.78 ± 0.04			
	8 weeks	0.70 ± 0.03*	-10.3%	0.73 ± 0.04*	-6.4%	0.26	0.13
	12 weeks	0.66 ± 0.03**	-15.4%	0.75 ± 0.05*	-6.3%	0.07	0.08
Fasting Glucose (*mmol/L*)	Baseline	5.49 ± 0.10		5.63 ± 0.11			
	8 weeks	5.31 ± 0.11	-3.3%	5.46 ± 0.10	-2.9%	0.45	0.42
	12 weeks	5.39 ± 0.10	-2.2%	5.41 ± 0.12*	-4.8%	0.85	0.92
Fasting Insulin (*pmol/L*)	Baseline	82.65 ± 8.33		110.43 ± 13.13			
	8 weeks	53.48 ± 4.58**	-35.2%	75.70 ± 10.42*	-31.0%	0.66	0.82
	12 weeks	60.42 ± 5.69**	-26.8%	88.90 ± 11.74*	-22.3%	0.59	0.74
HOMA^1^	Baseline	3.0 ± 0.4		4.1 ± 0.6			
	8 weeks	1.8 ± 0.2**	-38.9%	2.7 ± 0.4*	-32.6%	0.63	0.81
	12 weeks	2.0 ± 0.2*	-29.7%	3.0 ± 0.5	-24.9%	0.61	0.76
HbA1c *%*	Baseline	5.7 ± 0.1		6.0 ± 0.1			
	8 weeks	5.6 ± 0.1*	-1.9%	5.9 ± 0.1**	-2.7%	0.77	0.72
	12 weeks	5.6 ± 0.1**	-2.6%	5.8 ± 0.1**	-3.8%	0.82	0.87

*Different from baseline, *P *< 0.05

**Different from baseline, *P *< 0.01

^1^HOMA score calculated as follows: [(insulin (pmol/L)*glucose (mmol/L))/135].

^2^Comparing the values between arms using 1-sided unpaired t-test

^3^Adjusted for body weight, caloric intake, and carbohydrate intake

### Glucose, insulin, HbA1c, and HOMA score

Subjects in both arms exhibited decrease in fasting insulin concentrations and HbA1c at 8 and 12 weeks comparing to baseline (Table 3). With respect to fasting glucose, only subjects in the MED arm displayed reduction from baseline at 12 weeks. A reduction in HOMA score was observed in PED arm at both 8 weeks and 12 weeks, but in MED arm only at 8 weeks. No differences between arms were observed with respect to fasting insulin, fasting glucose, HOMA score, and HbA1c at both 8 and 12 weeks. Additional adjustment for calorie and carbohydrate intake, and body weight change for these variables did not change the findings (adjusted P values in Table 3).

### Metabolic syndrome variables and Framingham 10-year CVD risk score

At baseline, 23 subjects in the PED arm and 22 subjects in the MED arm met at least 3 of 5 criteria for MetS. The mean number of MetS criteria for the PED arm including only those subjects with MetS at baseline and only those completing 12 weeks was 3.81 ± 0.18 (n = 21), and 4.17 ± 0.19 (n = 18) for the MED arm. After 12 weeks on the trial, the number of MetS variables reduced to 2.95 ± 0.26 in the PED arm (*P *= 0.01), and to 3.56 ± 0.28 in the MED arm (*P *= 0.07). At the end of 12 weeks, 9 of the 21 subjects (43%) in PED arm no longer met criteria for MetS compared with only 4 of the 18 subjects (22%) in the MED arm. The calculated Framingham 10-year CVD risk score in subjects finishing all visits of the trial fell from 15.3 ± 2.5% at baseline to 9.6 ± 1.4% at 12 weeks in the PED arm (*P *< 0.001), and from 16.0 ± 3.0% at baseline to 13.1 ± 2.7% at 12 weeks for the MED arm (*P *= 0.011).

## Discussion

Due to the complex mechanistic underpinnings of MetS, strategies for reducing its incidence and consequences need to be similarly comprehensive and multi-factorial. Targeting multiple, chronically dysregulated signaling pathways at play in insulin resistance is likely to yield the greatest benefit [45] in the treatment of MetS. To our knowledge, this is the first intervention study to successfully demonstrate the concept of modifying cardiometabolic risk factors by combining a modified Mediterranean-style, low glycemic load dietary pattern, and regular exercise, with diverse phytochemicals targeting multiple signaling abnormalities of MetS. A greater than 2-fold improvement in total cholesterol, non-HDL cholesterol, TG, cholesterol/HDL, and TG/HDL were observed with the addition of soy protein, phytosterols, hops RIAA, and acacia PAC to a modified Mediterranean-style low glycemic load diet and exercise program. Increase in HDL and decrease in LDL and VLDL particle numbers were seen only in the PED arm. Also, diastolic BP fell only in the PED arm. Furthermore, by 12 weeks, almost double the number of subjects experienced a net resolution of MetS in the PED group compared with MED group, and the PED group experienced almost twice the reduction in the Framingham 10-year CVD risk score as the MED group. Overall, CVD risk was reduced to a greater degree in the subjects supplemented with phytochemicals relative to the subjects on MED alone.

Previously [37], we compared a calorically-restricted version of the diet used in this study supplemented with soy protein and phytosterols to the frequently-studied AHA Step I low-fat diet in postmenopausal hypercholesterolemic women. We demonstrated that this targeted phytochemical, supplemented diet produced greater benefits than the AHA diet alone. The protocol for the current trial differed from that of our previous study in 2 key aspects: (i) subjects in both study arms were instructed to consume their diet until satisfied, and (ii) 2 additional supplemental phytochemicals were provided to target the underlying inflammatory mechanisms of MetS. The modified Mediterranean-style, low glycemic load diet by itself is an effective approach to weight loss and CVD risk reduction [46]. Our findings suggest that the addition of the soy protein, phytosterols, in combination with targeted phytochemicals was responsible for the more favorable CVD risk profile outcome in the PED group. This observation could not be attributed to greater weight loss in the PED group, since the extent of weight loss was similar on both arms. A recent review by Volek and Feinman reported that dietary carbohydrate may affect various cardiometabolic factors [10]. In our analysis, we adjusted for calorie and carbohydrate intake, and body weight change, and found that the observed differences between arms remained unaffected (Table 3). Likewise, the glycemic loads of the diets and the levels of exercise did not differ between arms. Although provision of the powdered beverage may have simplified meal planning, and in turn, enhanced dietary compliance and convenience, an effect similar to that reported by Noakes et al. [47], again weight loss did not differ. Finally, although only subjects in the PED arm experienced a decrease in hunger levels in the period between the evening meal and bedtime, which might have reduced excessive, nighttime snacking, differences in intake should have been reflected in differential body weight loss and recorded caloric intake, neither of which occurred.

RIAA is a modified hop extract that has been used in beer for flavoring, foam stability and bittering for decades, and is prized for its chemical stability. RIAA is derived from hop cones in a process that first involves extraction of whole hops with supercritical CO_2_, yielding an extract containing a mixture of alpha acids, beta acids and hop oils [48,49]. The alpha acids are differentially isolated, isomerized, and reduced to form RIAA. The *Acacia nilotica *tree grows naturally in many arid climates in Australia, Africa, and India and the young trees are a food source for many animals including cattle [50-52]. The young leaves have been fried and consumed like other leafy vegetables. The bark is ingested traditionally as a hot beverage and in a more concentrated form as a folk medicine for various ailments including colds, diarrhea, tuberculosis and leprosy [53]. The rationale for inclusion of the RIAA/PAC tablet was based on results of in vitro screening studies employing insulin resistant 3T3-L1 adipocytes, *db/db *mouse diabetes studies, and a pilot clinical trial (unpublished results); all consistently showing improvement in insulin sensitivity. Briefly, the RIAA/PAC combination displayed lipogenic and anti-inflammatory activity in murine 3T3-L1 adipocytes stimulated with TNFα. Both agents inhibited TNFα-stimulated IL-6 secretion and improved adiponectin secretion. Individually and in various combinations these compounds also demonstrated favorable modulation of the activity of proteins and kinases implicated in insulin signaling such as PI3K, GSK-3, AKT, PKC and c-Jun N-terminal kinase (manuscript in preparation). Other groups have reported that feeding of isohumulone, which is structurally similar to RIAA, to C57BL/6N mice reduced plasma TG and free fatty acid levels [54]. Additional animal and clinical studies with isohumulone have revealed that: (i) diabetic KK-Ay mice had reduced plasma glucose, TG, and free fatty acid levels, 65.3, 62.6, and 73.1%, respectively; (ii) C57BL/6N mice fed a high fat diet showed improved glucose tolerance and reduced insulin resistance; and (iii) a double-blind, placebo-controlled pilot study on diabetic subjects suggested that isohumulones decreased blood glucose and HbA1c levels by 10.1 and 6.4%, respectively, after 8 weeks [55]. We chose RIAA over IAA due to RIAA's greater chemical stability [48], potent in vitro anti-inflammatory activity in 3T3-L1 adipocytes. In our pilot clinical trial a combination of RIAA and PAC in tablet form significantly improved LDL, TG and TG/HDL in subjects with MetS.

The results of the present study, in agreement with similar studies reported by others [25-28], confirm the benefits of lifestyle intervention consisting of a phytochemical rich, low glycemic load diet and a moderate aerobic exercise regimen in subjects with MetS. For example, Jenkins et al. [56] found reductions in lipid variables with an *ad libitum *low glycemic load diet ("portfolio diet") in subjects with high TG at baseline. The average reductions reported by Jenkins et al. in total cholesterol, LDL and TG over one month were almost identical to those noted in the MED arm of the present study over 8 weeks (8.8% vs. 8.7%; 9.1% vs. 9.8%; and 19.3% vs. 22.3%, respectively). Also, similar to the results reported by Jenkins et al. [56], no changes in HDL were noted in MED subjects in the present study. Lastly, in both arms of the present trial, we found that subjects' food cravings fell and satiety increased with institution of the low glycemic load diet and energy intake fell despite the lack of instructions to limit caloric intake. Esposito et al. reported that 48% of subjects with MetS instructed to follow a Mediterranean-style (not necessarily low glycemic) diet over 2 years no longer met 3 or more criteria for MetS [57]. In the Diabetes Prevention Program Trial, lifestyle modification led to 38% resolution of MetS after 3 years [58]. In contrast, only minimal resolution of MetS was seen in the Diabetes Prevention Program Trial after one year with either metformin or lifestyle management [58]. Our study suggests that by complementing a therapeutic lifestyle program with a soy- and phytosterol-based powdered beverage and tablets containing hops RIAA and acacia PAC, subjects can improve to a similar magnitude (43%) attained at 2 and 3 years in just 12 weeks.

Most therapeutic treatments for hypercholesterolemia focus on achieving LDL goals recommended by NCEP. However, the NHANES 2003–2004 showed that despite better control of LDL, other lipid risk factors remained suboptimal in many US adults, particularly among those with CVD, diabetes, or MetS [59]. Non-HDL cholesterol, a stronger predictor of CVD and mortality risk than LDL [60-62], has now been added by the NCEP Adult Treatment Panel III as a secondary target of therapy [63]. In addition, because apo B indicates the total number of atherogenic lipoprotein particles and apo A-I, a major lipoprotein in HDL, has a critical role in reverse cholesterol transport, the apo B/apo A-I (as well as apo B concentration) has been proposed as a risk factor for CVD. Increasing evidence from multiple studies has repeatedly shown that the apo B/apo A-I predicts cardiovascular risk – the lower the ratio, the lower is the risk – and is a better marker than LDL and lipid ratios [64-71]. In the InterHeart study, the apo B/apo A-I was the strongest determinant of MI risk, even higher than smoking; the OR of top vs. lowest decile was 4.73 [72]. The authors state the apo B/apo A-I might be the best marker of the balance of atherogenic and antiatherogenic particles. Subjects in both PED and MED arms showed significant reduction in non-HDL cholesterol and apo B/apo A-I at 8 weeks compared to baseline, but only the PED arm showed continued reduction in the ratio at 12 weeks. These data suggest further cardiovascular benefit from the added phytochemicals.

## Conclusion

The worldwide prevalence and multi-factorial nature of MetS do not reasonably support a pharmacologic approach for treatment or prevention. Fortunately, lifestyle modification including dietary alteration has demonstrated success in correcting metabolic abnormalities associated with the development of type 2 diabetes and CVD. The present study provides evidence that supplementation of a modified Mediterranean-style, low glycemic load diet with a combination of phytochemicals addressing multiple inflammatory and insulin signaling pathways simultaneously may be a novel, effective means to managing MetS. This comprehensive, supplemented lifestyle program represents a potentially powerful approach to the management of at risk individuals with MetS and hypercholesterolemia.

## List of abbreviations

AHA: American Heart Association; Akt: protein kinase B; apo: apolipoprotein; BMI: body mass index; BP: blood pressure; CVD: cardiovascular disease; GSK-3: glycogen synthase kinase-3; HbA1c: hemoglobin A1c; HOMA: homeostatic model assessment; MED: modified Mediterranean-style low glycemic load diet; MetS: metabolic syndrome; NCEP: National Cholesterol Education Program; PAC: proanthocyanidin; PED: phytochemical – supplemented MED; PI3K: phosphoinositol 3-kinase; PKC: protein kinase C; RIAA: *rho *iso-alpha acids; TG: triglycerides; TNFα: tumor necrosis factor alpha.

## Competing interests

The study was funded by MetaProteomics, LLC, a subsidiary of Metagenics, Inc that manufactures the commercial medical food, UltraMeal^® ^Plus, for licensed healthcare professionals. The combination nutraceutical referred to in the manuscript is a commercial product (Insinase™) developed by Metagenics, Inc. All authors are employees of MetaProteomics.

## Authors' contributions

All authors participated in the concept and design of the study, and contribute to manuscript preparation. RHL was the principle investigator. RHL, BS, JJL, and DMM conducted the intervention study. DMM and BS provided dietary counseling. RHL, GD, and MLT performed the statistical analysis. All authors read and approved the final manuscript.

## Supplementary Material

Additional file 1 **List of permitted foods and beverages, serving sizes and recipes.** Click here for file

Additional file 2 **Macronutrient profile of the soy and phytosterol-based powdered beverage.** Click here for file
