# Supplementary material for: Enhancement of a modified Mediterranean-style, low glycemic load diet with specific phytochemicals improves cardiometabolic risk factors in subjects with metabolic syndrome and hypercholesterolemia in a randomized trial
Source: Nutr Metab (Lond). 2008 Nov 4;5:29. doi: 10.1186/1743-7075-5-29 (PMC2588603; doi:10.1186/1743-7075-5-29)
Supplement: Additional file 1 — List of permitted foods and beverages, serving sizes and recipes. [file 1743-7075-5-29-S1.pdf]

Additional file 1: List of Permitted Foods and Beverages, Serving Sizes and Recipes

Food Category	Average Serving Size	Daily Recommendation	Examples
Legumes	½ cup	At least 1 serving	Beans, hummus, green peas
Low GI* (<55) Vegetables	½ cup	At least 3 servings	Broccoli, celery, cabbage, spinach, mushrooms, tomatoes, sea vegetables, fresh juices made from acceptable vegetables
Moderate GI (55-70) Vegetables	½ cup	Only 1 serving	Beets, sweet potatoes, carrots
Concentrated Protein Sources		Unlimited	Eggs , fish, poultry, tofu, low-fat cottage cheese, low-fat mozzarella
Fruits	Specific quantities for each fruit to equal 80 kcal	2-3 servings	Apple, blueberries, cherries, grapes, orange
Nuts and Seeds	Approx. 1 scant handful or 2 TBSP nut butter	1 serving	Peanut butter, almonds, hazelnuts, walnuts
Oils	1 teaspoon or as indicated	4 servings	Flaxseed oil, olive oil, ripe green olives, avocado
Dairy	6 oz. or as indicated	(Optional)	Plain low-fat yogurt, 1% milk, soymilk (unsweetened)
Beverages		Unlimited	Decaffeinated, herbal, or green tea; decaffeinated coffee; water
		Limited	1 glass of red wine (4 oz.), 1-2 cups (8 oz.) of caffeinated coffee
Condiments		Unlimited	Cinnamon, mustard, soy sauce, flavored extracts
Allowable sweeteners			Agave nectar, stevia

To be eliminated from diet	Examples
Trans fatty acids	Partially hydrogenated vegetable oil
Artificial sweeteners	Splenda® (sucralose), NutraSweet® (aspartame)
High GI fruits and vegetables	White potatoes, corn, bananas
Simple sugars	High fructose corn syrup, fruit juice, table sugar (sucrose), evaporated cane juice, honey, maple syrup

*Glycemic Index
