# Supplementary material for: Enhancement of a modified Mediterranean-style, low glycemic load diet with specific phytochemicals improves cardiometabolic risk factors in subjects with metabolic syndrome and hypercholesterolemia in a randomized trial
Source: Nutr Metab (Lond). 2008 Nov 4;5:29. doi: 10.1186/1743-7075-5-29 (PMC2588603; doi:10.1186/1743-7075-5-29)
Supplement: Additional file 2 — Macronutrient profile of the soy and phytosterol-based powdered beverage. [file 1743-7075-5-29-S2.pdf]

Additional file 2: Macronutrient profile of the soy and phytosterol-based powdered beverage[1]

**Nutrient**	**Amount per serving**	**Recommended Daily Intake (%)**
Total Fat	2 g	3
Cholesterol	0 mg	0
Total Carbohydrate	24g	8
Dietary Fiber	4 g	16
Soy Protein	15 g	30
Phytosterols[2]<br>(β-sitosterol 40-58%;<br>campesterol 20-30%;<br>stigmasterol 14-22%;<br>brassicasterol 0-6%;<br>and sitostanol 0-5%)	2 g	

[1]Calories per serving, 160; [2]Recommended daily intake not established.
